# Expecting Rejection: Understanding the Minority Stress Experiences of Transgender and Gender-Nonconforming Individuals

**DOI:** 10.1089/trgh.2016.0012

**Published:** 2016-08-01

**Authors:** Brian A. Rood, Sari L. Reisner, Francisco I. Surace, Jae A. Puckett, Meredith R. Maroney, David W. Pantalone

**Affiliations:** ^1^ Department of Psychology, Augsburg College, Minneapolis, Minnesota.; ^2^ Division of General Pediatrics, Boston Children's Hospital/Harvard Medical School, Boston, Massachusetts.; ^3^ The Fenway Institute, Fenway Health, Boston, Massachusetts.; ^4^ Department of Epidemiology, Harvard T.H. Chan School of Public Health, Boston, Massachusetts.; ^5^ Department of Psychology, University of Massachusetts, Boston, Massachusetts.; ^6^ Department of Psychology, University of South Dakota, Vermillion, South Dakota.; ^7^ Department of Counseling and School Psychology, University of Massachusetts, Boston, Massachusetts.

**Keywords:** expecting rejection, gender, minority stress, stigma, transgender

## Abstract

**Purpose:** Transgender and gender-nonconforming (TGNC) individuals often are the target of enacted or external (i.e., distal) experiences of stigma, discrimination, and violence, which are linked to adverse health, particularly psychological distress. There is limited research, however, examining felt or internal (i.e., proximal) stressors faced by TGNC individuals. This study sought to examine one type of internal stressor, *expecting rejection*, and aimed to (1) identify how and to what extent rejection expectations operate day-to-day for TGNC individuals and (2) explore how TGNC individuals respond to expectations of rejection.

**Methods:** In-depth interviews were conducted with 30 participants from 2014 to 2015 who identified as TGNC (mean age=30.4; 60% people of color); data were analyzed using a consensual qualitative research method.

**Results:** Four thematic categories emerged about expecting rejection: (1) where to expect rejection; (2) thoughts and feelings associated with expectations of rejection; (3) coping strategies used to manage the expectation of rejection; and (4) the intersection of race and ethnicity with rejection expectations.

**Conclusion:** Findings from this study suggest that expecting rejection is a frequent and salient internal stressor for TGNC individuals. We discuss the psychological and cumulative potential health impact of minority stress, and the applicability of Meyer's Minority Stress Model. Therapeutic interventions are needed to address the specific cognitive, emotional, and behavioral responses TGNC individuals experience as a result of the stress associated with expecting rejection, including fear, anxiety, and situational avoidance.

## Introduction

Transgender and gender nonconforming (TGNC) individuals have a current gender identity or expression that differs from their assigned sex at birth. Although research investigations that focus specifically on the health and well-being of TGNC individuals remain limited, peer-reviewed published articles have documented the pervasiveness with which TGNC people face enacted experiences of stigma, discrimination, and victimization.^1–4^ Prevalence estimates of discrimination among TGNC individuals are shown to be extraordinarily high, exceeding 60% in several published studies.^5,6^ Similarly, estimates for victimization are commonly greater than 40% for TGNC people.^7,8^ Emerging research also has highlighted an association between enacted stressors and indicators of negative mental and physical health. For example, TGNC individuals who reported having experienced physical or sexual abuse, compared to those who did not, are significantly more likely to report a history of suicidal ideation and suicide attempts.^9,10^ Likewise, experiences of gender-related discrimination are shown to be significantly associated with elevated levels of psychological distress for TGNC individuals.^11^ Thus, enacted stressors appear to be detrimental to the health of TGNC individuals and continued and ongoing research, particularly longitudinal studies examining the relationship of such stressors to health over time, is needed.

Enacted stressors, however, represent only the *external* processes and experiences faced by TGNC individuals. TGNC individuals likely experience *internal* stressors and processes in response to these and other external stressors. Consistent with the theory and empirical research underlying Meyer's Minority Stress Model, internal or *proximal stressors* are considered more subjective and related to self-identity.^12–15^ According to the model, the three specific proximal stressors recognized are as follows: (1) identity concealment, (2) internalized stigma, and (3) expectations of rejection.

Expecting rejection—the focus of the present article—is described in the literature as a form of felt stigma, which is understood as an individual's knowledge of society's stance toward nonmajority individuals, and expectations regarding the likelihood of stigma being enacted in a given situation as a result of having a minority status, for example, for sexual and gender minority individuals.^16^ Notably, research has demonstrated that having a dual minority status (e.g., being a person of color who is also a sexual minority) can further complicate and heighten experiences of enacted stigma, which has implications for expecting rejection, especially among individuals who represent more than one marginalized identity.^17^

There is a moderate amount of published data to show how expecting rejection operates in the lives of cisgender (i.e., nontransgender or someone whose gender identity matches what is typically associated with someone of their sex assigned at birth) sexual minority individuals.^18–20^ Yet, published research that demonstrates how expecting rejection is experienced by TGNC individuals remains scarce.

Based on a review of the extant literature, only a few published studies have empirically investigated how expecting rejection might operate in the lives of TGNC individuals. Bockting et al. surveyed 1,093 transgender individuals and found that the expectation of rejection (i.e., felt stigma) was positively associated with psychological distress, and negatively associated with levels of outness.^11^ Gamarel et al. examined relationship stigma—real or anticipated feelings of negative judgment from others as a result of one's romantic relationship being socially devalued—among couples comprising cisgender men and transgender women.^21^ They found that higher levels of reported relationship stigma were associated with increased odds (adjusted odds ratio=1.13) of reporting clinically significant depressive distress.^21^

Thus, there is support for the notion that TGNC individuals might expect rejection specific to their gender identity, and preliminary data appear to support an association between expecting rejection and psychological distress.^11^ However, the internal experience of expecting rejection is not well understood. Specifically, we do not know how TGNC individuals perceive and assign meaning to this expectation and how they respond emotionally. Examining the internal process through which TGNC individuals might expect rejection would offer a more informed perspective regarding the noted association between experiences of stigma and adverse mental health, including implications for future points of therapeutic intervention.

Two recently published studies have also investigated variations of expecting rejection among TGNC individuals. In one study, investigators examined specific situations or environments that TGNC individuals might avoid, based on the fear of being outed against their wishes. With a sample of 889 TGNC individuals, 38.8% reported avoiding public restrooms, 38.4% reported avoiding gyms, 29.8% reported avoiding clothing shops, and 24.0% reported avoiding public transportation.^22^ Although these findings are helpful in identifying situations where TGNC individuals may expect rejection, they do not describe why or how the expectation might manifest, or how TGNC individuals may respond to the perceived threat. Increasing knowledge in this area would aid researchers in identifying how to target and effectively address areas perceived as unsafe (e.g., through stigma reduction interventions).

In the second study, investigators examined the association between employment status, coping strategies, and internalized transphobia and transgender stigma among 55 TGNC individuals.^23^ The sample consisted of 24 transgender women (male-to-female), 20 transgender men (female-to-male), seven genderqueer or gender fluid individuals (who do not situate themselves within the gender binary), and four individuals who were either undecided or did not indicate their gender. The study demonstrated that higher levels of effective coping strategies (e.g., coping with work- and mental health-related stigma) were associated with lower levels of internal and external stigma. This study provides preliminary information about the role of coping in managing proximal stressors, particularly as a moderating variable, which is an important consideration because coping strategies related to proximal stressors for TGNC individuals are relatively unexplored and are a key component of Meyer's model that warrants further investigation.^12,13^

Given the relative lack of investigations specific to the proximal stress experiences of TGNC individuals, and expecting rejection in particular, this study sought to comprehensively understand these experiences for TGNC individuals through in-depth interviews. Specifically, this study aimed to (1) identify how, and to what extent, expecting rejection operates day-to-day for TGNC individuals and (2) explore how TGNC individuals respond to expectations of rejection. Given the space constraints of a single article, we focus, in this study, on maladaptive or problematic coping strategies and experiences reported by the participants; we will present data on adaptive or resilient coping strategies in a separate article, to give that aspect of the participant's narratives the space they deserve.

## Methods

### Procedures

#### Ethical considerations

The Institutional Review Board at Suffolk University approved the research study. All participants electronically signed consent forms before participating. No adverse events were reported to investigators.

#### Research team

The research team consisted of six members: one team member was a postdoctoral researcher (White, genderqueer); two were doctoral students in clinical psychology (both Latino, cisgender men); and two were Master's-level psychology students (both White, one a cisgender woman and the other transmasculine). The team was supervised by a clinical psychologist and university professor (White, cisgender man) with expertise in sexual and gender minority health, and experience in qualitative research methods.

#### Recruiting interviewees

Participants were recruited through message boards, Listservs, and social networking sites on the Internet. Online recruitment was important for this study because research suggests that the Internet is a particularly viable source of networking and information gathering for transgender individuals.^24^ Recruitment letters and online flyers were sent electronically to websites with a predominant transgender audience. The materials contained basic information about the nature of the research study, the investigators, and a link to the web address for the online study. Study consultants also posted a link to the online study through private social networking groups (e.g., in Yahoo! and Facebook).

After reviewing the recruitment materials, interested potential participants were directed to a secure area of SurveyMonkey.com where they could complete the eligibility screener. If eligible and willing to participate, participants then completed an online questionnaire that included measures of mental and physical health, health behaviors, and social support. This represented phase one of the research study. Following the completion of the questionnaire, participants were asked to indicate if they would like to participate, at a later date, in an in-depth interview, which represented phase two of the research study. From the list of individuals willing to take part in the interview, we randomly selected participants and used purposive sampling until we reached an even number of individuals who were male assigned and female assigned at birth.

The 30 participants in this study cited the following sources when asked to specify how they were recruited: transgender social networking sites (11 participants), email from a friend/colleague (7 participants), Facebook group/friends (5 participants), transgender support websites (4 participants), closed online support group (1 participant), Tumblr (1 participant), and online transgender message boards (1 participant).

#### Eligibility criteria

Eligible participants were individuals who (1) resided in the United States; (2) identified as “transgender” or “noncisgender/other”; (3) were between the ages of 25–40 years; and (4) have begun the gender affirmation/transition process by dressing much/most of the time as their identified gender for at least the past 6 months.

The sample was restricted geographically (U.S.-based) and with regard to age. The restricted age range was selected to include individuals who are beyond emerging adulthood, yet not quite at midlife. Limiting the geographic region and ages of participants was implemented to minimize the presence of life stressors that are unique to age (e.g., emerging adults who might be living independently for the first time), generation (e.g., older adults who lived through periods of relatively greater social stigma regarding LGBT individuals), or nationality (e.g., U.S. legislation compared with European legislation regarding LGBT rights).

In addition, participants were required to have affirmed their gender through the way they dressed because this study assessed ways in which their environment (e.g., friends, family, and employment/school) might have enacted stigma in response to their gender presentation. Therefore, TGNC individuals who were not affirming their gender identity, particularly in ways that were not visible to their external environment, might not have experienced potential stigma/stress and, thus, were not eligible, given the aims of this study.

#### The interview

The principal investigator (first author) developed the interview protocol in collaboration with several community-based researchers, experts in qualitative methods, and experts in sexual and gender minority health. Given that this study was designed to more thoroughly understand how TGNC individuals experience proximal stressors, the questions for the interview were developed to assess for specific constructs represented in Meyer's Minority Stress Model.^12,13^ The interview protocol included questions about, for example, expectations of rejection, experiences of hypervigilance, identity concealment, passing, and the intersection of race/ethnicity with gender identity.

Initial interview questions were more general, in an effort to establish rapport. Subsequent questions focused on more sensitive content. Although the interview protocol consisted of specific questions, discussion of each content area began with an open-ended question so that each participant could detail (1) if the experience was personally relevant, (2) what occurred, (3) what the associated thoughts and feelings were, and (4) how they responded to the experience.

The first author (Latino, cisgender man) conducted all interviews online through Skype. Skype offers encryption for voice conversations and is regarded to be among the most secure of Voice over Internet Protocol providers. Each participant was provided access to a participant Skype account with a unique password; participants did not use a personal account that might have included personal identifiers. Before the start of each interview, the interviewer and participant reviewed the purpose and voluntary nature of the research study and the informed consent document the participant had previously signed electronically.

During the interview, the video option on the interviewer's Skype account was turned off; therefore, no video transmission of the participant was received. The audio, however, was recorded using a digital audio recorder placed next to the computer speaker. Interviews ranged from 37 to 94 min in length (mean 62.2, standard deviation 11.9). Following the interview, participants were provided with a list of transgender resources and sent a $30 gift card electronically.

#### Data analysis

All interviews were transcribed by undergraduate research assistants and checked for accuracy by two independent reviewers. All identifying information (all proper nouns, such as names and places) was removed and identification numbers were assigned to ensure confidentiality. Audio recordings were deleted once transcription was finalized.

Data analysis carefully followed the consensual qualitative research (CQR) approach.^25^ To begin coding the data, the research team first developed a tentative list of domains, based on a review of the literature and primary questions in the interview protocol. The domains represent general topic areas that were covered in the interview guide. The team then took the initial domain list and independently coded the same four transcripts. In this process, the domain list was tested by applying it to the transcripts to determine how well the domains “fit” these data. Consistent with the CQR methodology, the domain list was independently modified (e.g., adding, removing, or combining domains) based on the topic areas that emerged from transcripts.^25^ After this process, the team met to compare notes and discuss changes to the domains, until a stable list was decided upon.

After developing the consensual domain list, team members coded the remaining transcripts in pairs. For each transcript, the two members coded independently and then met together to compare codes and consensually assign domains to the data. For each coding pair, there was a third team member identified to serve as an auditor. The auditor reviewed the coded transcript produced by the team of two, and assisted the pair in reaching consensus for any disagreements or discrepancies.

After coding all 30 transcripts, the team reviewed each transcript for core ideas. Core ideas represent clear and concise summaries of each piece of data (i.e., the participant's narrative). The function of constructing core ideas is to concisely label each piece of data, which enables more effective comparisons across participants in the later cross-analysis.^25^ After constructing core ideas for the transcripts, the team met, discussed the data, and reviewed the core ideas until we reached consensus.

For the cross-analysis, the team examined the core ideas represented within each of the domains across all participants. In this stage of analysis, the team consensually grouped core ideas into common themes and constructed categories and subcategories within each domain. Consistent with the CQR methodology, categories and subcategories were labeled as *general* if they applied to 29 to 30 cases (i.e., participants), *typical* if they applied to 16 to 28 cases, and *variant* if they applied to 2 to 15 cases.^25^ Through team discussions, consensual revisions were made to study data until results were finalized.

### Measures

#### Demographics

Participants were asked for their U.S. zip code, sex assigned at birth, gender identity, sexual orientation, age, relationship status, level of education, race/ethnicity, and engagement in different gender affirmation activities (e.g., *Have you dressed much/most of the time in your identified gender for at least the past 6 months? Have you disclosed to friends and family about your gender identity? Have you participated in hormone therapy?*).

#### Interview guide

The interview guide included questions to assess the experience of expecting rejection. For example: “Have you ever expected that you might experience discrimination because you identify as transgender? [If yes] Please tell me about an experience that stands out to you the most when you expected to experience discrimination.” (See Appendix.)

## Results

### Sample characteristics

For the total sample, the mean age was 30.4 years and the majority of participants endorsed a non-White race/ethnicity (60.0%). The majority of participants identified as women (26.7%) or as transmen (female-to-male; 23.3%). Forty-three percent of participants identified their sexual orientation as queer, and the majority of participants either graduated from college (43.3%) or took college-level coursework (26.7%). Most participants reported engaging in masculinizing or feminizing hormone therapy (76.6%), considered some form of gender reassignment surgery (80%), and told people at work or school about their affirmed gender (86.7%). Participants resided throughout different geographic regions of the United States. Complete sample characteristics are presented in Table 1.

**Table 1.** **Sample Characteristics**

Characteristic	*n*	% of sample
Age (M, SD)	30	30.4 years (6.1)
Sex assigned at birth		
Female	15	50.0
Male	15	50.0
Race/ethnicity		
White	12	40.0
Biracial/multiracial	10	33.3
Asian/Asian American	3	10.0
Black/African American	3	10.0
Latino/Hispanic	2	6.7
Gender identity		
Female/woman	8	26.7
Transmale/Transman (FTM)	7	23.3
Transfemale/Transwoman (MTF)	4	13.3
Male/man	4	13.3
Genderqueer	3	10.0
Gender fluid	1	3.3
Masculine	1	3.3
Transboi	1	3.3
Transsexual	1	3.3
Sexual orientation identity		
Queer	13	43.3
Lesbian/gay	5	16.7
Heterosexual/straight	4	13.3
Pansexual	4	13.3
Bisexual	3	10.0
Asexual	1	3.3
Relationship status		
Single	9	30.0
Partnered	7	23.3
Married	7	23.3
In an open relationship	3	10.0
Engaged	2	6.7
In many open relationships	1	3.3
Nonromantic coparent relationship	1	3.3
Educational attainment		
College graduate	13	43.3
Some college	8	26.7
Graduate level education	5	16.7
High school graduate/GED	4	13.3
Have participated in the following gender affirmation activities		
Told friends or family about affirmed gender	30	100.0
Told people at work or school about affirmed gender	26	86.7
Considered some form of GRS	24	80.0
Hormone therapy	23	76.7
Completed some form of GRS	10	33.3
United States geographic region		
West	11	36.7
South	9	30.0
Midwest	7	23.3
Northeast	3	10.0

FTM, female-to-male; GED, General Educational Development certificate; GRS, gender reassignment surgery; MTF, male-to-female.

### Qualitative results

Nine domains emerged following the consensual coding process (Table 2). Within the Expecting Rejection domain, there were four distinct categories: (1) where to expect rejection (with four subcategories); (2) thoughts and feelings associated with expecting rejection (with six subcategories); (3) coping strategies used to manage the expectation of rejection (with three subcategories); and (4) the intersection of race and ethnicity with expecting rejection (with two subcategories). Categories and subcategories applying to only one case were excluded from the results. All domain categories and subcategories are presented in Table 3. Next, themes are presented and discussed with accompanying illustrative quotes, along with self-reported age, race/ethnicity, and gender identity of the participant who shared the quote.

**Table 2.** **Domain List**

1. Expecting rejection
2. Concealing or hiding gender identity
3. Passing
4. Negative social messages
5. Intersection of race/ethnicity and gender-related stress
6. General coping with gender-related stress
7. Sources of support
8. Resilience
9. Positive message to share with others

**Table 3.** **Categories, Subcategories, Illustrative Core Ideas, and Frequencies Regarding the Expecting Rejection Domain (*N*=30)**

Domain/category/subcategory	Illustrative core idea	Frequency
Expecting rejection		
(1) Where to expect rejection		General (30)
(a) Could happen in situations that include gender markers or a clear gender binary system	Public restrooms; spaces that ask for personal identification; healthcare settings	General (29)
(b) Could happen anytime when out in public spaces or when there is the potential to meet new people	Anywhere I go/everyday/it is part of being transgender or gender nonconforming; when I am around new or unfamiliar people/environments; when in crowds	Typical (27)
(c) Could happen when around people who know me or with whom I plan to interact	Employment/work settings; when with family; when around people who knew me pretransition	Typical (20)
(d) Could happen if/when I do not pass	When I was not passing well earlier in my transition; when I was not on hormone replacement therapy	Variant (5)
(2) Thoughts and feelings associated with expecting rejection		General (30)
(a) Anxious/stressed/nervous	Experiencing anxiety/nervous; experiencing general stress; feeling overwhelmed	General (30)
(b) Fearful/worried about safety/hyperaware	Experiencing fear or terror; worrying about personal safety or violence; feeling on alert or on guard	General (29)
(c) Depressed/self-loathing/my fault	Experiencing sadness/depression; picking self-apart/self-denigrating; feeling like a burden/it is my fault	Typical (17)
(d) Angry/frustrated with the situation and others	Feeling anger/irritability/rage; feeling frustrated; feeling like it is unfair	Typical (17)
(e) Not supported/ignored/rejected	Feeling ignored or invisible; thinking about the lack of support; feeling rejected by others	Variant (12)
(f) Physically exhausted by the end of the day	Feeling physically exhausted by the end of the day; feeling tightness in body; feeling shaky	Variant (7)
(3) Coping strategies used to manage the expectation of rejection		Typical (26)
(a) Avoidance/escape	Avoiding specific situations and people; hiding when in the situation; escaping the situation	Typical (17)
(b) Substance use	Alcohol; smoking cigarettes; Marijuana use	Typical (17)
(c) Cognitive/emotional coping strategies	Ruminating/thinking about the situation; feeling angry; minimizing the severity of the situation	Variant (7)
(4) Intersections of race and ethnicity with expecting rejection		Typical (20)
(a) Being a person of color increases the expectation of rejection/helps you to prepare for the rejection	Expect more rejection if you are a person of color; being a person of color prepares you for the possible rejection; in addition to my gender, being a person of color makes me stand out and puts me on guard	Typical (19)
(b) Being White comes with privileges/decreases the expectation of rejection	Being White or perceived as White has privileges, and lowers the expectation of rejection	Variant (12)

### Where to expect rejection

#### Could happen anytime when out in public spaces or when there is the potential to meet new people

Participants specified that they expected rejection anytime they left home and entered a public space. Examples of different public spaces included grocery stores, restaurants, and hotels.

Really, it could be anywhere, you know? Those kinds of situations pop up where you least expect them. I mean, they're going to happen where you do expect them—you know, when you're walking out on the street or going to the grocery store and that mother of four is looking at you like you're going to hurt her children just because you dress differently. But, you know, it could pop up at you and it just snaps you back to how unusual your situation is, and it takes away from just living life normally. (27, White, female/woman)

Notably, participants identified spaces that, historically, are regarded as transphobic, specifically, rural and conservative geographic areas, and religious settings. Yet, participants also explained that transindividuals might expect rejection in LGBTQ-identified spaces.

I would oddly say pride, and events where it's LGBT run or friendly. A lot of our gay brothers and sisters do not want to recognize that we are who we are, and so I choose to be stealth in those environments. I do notice that I get along with girls, just because that's my nature, but the lesbians seem very stand-offish. They don't want to make eye contact with me and I get it, but at the same time it's like, “You're missing out on the fundamental point here! I'm a part of this community and I should feel welcome in this community, but I'm not sometimes.” (34, Black, male/man)

Overall, participants noted that expecting rejection in most environments is almost inevitable and a fundamental part of being TGNC identified.

There is the knowledge that you're going to walk into places and you will get treated differently; you will get looked at differently. It's not like it might not happen. It's not like it might happen. It's going to happen at least at some point every day. (40, White, gender fluid)

Oh, yes! Pretty much every day, every experience I go to, that [expecting rejection] is a big concern for me. (30, multiracial, male-to-female [MTF])

#### Could happen in situations that include gender markers or a clear gender binary system

Overwhelmingly, participants detailed that they expected rejection in spaces where they must interact with others in a way that made explicit their gender identity. The most frequently identified space was public restrooms and, similarly, locker/fitting rooms and medical visits.

There's definitely discomfort using public bathrooms… There's definitely a lot of anxiety. Sometimes I can just waltz into whatever bathroom that I want, super confidently, and then a lot of times I can't do that. I'll just be dodging eyes, not looking at people, so they won't be confrontational. I've never had any super-confrontational experiences in public bathrooms but I know a lot of people who have, and I can still feel looks sometimes, which I think could escalate, and that's what I'm worried about. (25, multiracial, genderqueer)

Participants noted specific concerns about using public restrooms that are associated with the expectation of rejection.

… and then of course there are always restrooms, which are, let's call them, “nightmarish,” for lack of a nicer term. I do everything possible to avoid using the restroom in public. Going into the bathroom, you're always worried that someone's gonna harass you or call you out about something, or say something mean or attack you, or do some other horrible thing. I've personally not been physically attacked ever in a bathroom but I've definitely had people give me weird looks a lot, and I've had people say to me that I was in the wrong bathroom. Bathrooms are always stressful. I much prefer to use private bathrooms… because then I don't have to deal with someone else coming in, bothering me, and harassing me. (30, multiracial, MTF)

In addition, participants were outspoken about experiencing stress in situations that required legal identification, for example, airport security or being stopped by the police.

Yeah, so, at the airport, for instance, because my passport and everything reads female, and my name is obviously female, immediately I'm nervous about that. When they have to look at the passport and at your fingerprints, or whatever. These kinds of situations make me very nervous and I expect that there will be problems, and that's what makes me uncomfortable. (32, White, female-to-male [FTM])

#### Could happen when around people who know me or with whom I plan to interact

Although participants frequently noted that they could reduce the stress associated with expecting rejection by avoiding specific places and contexts, they explained that certain contexts were not so easily avoided because they had ties to the people and the place. Most frequently, participants cited work/employment as a context in which they expect rejection.

It [expecting rejection] is why I am not at all out about my gender at work… There is no doubt that if I were open about being gender fluid, it would not only be the end of my career, but there would be a lot of personal repercussions as well… There's a definite fear that I could be found out at any time, and there's also anger—because it's dehumanizing. (40, White, gender fluid)

In addition, participants noted that family and people who knew the participants before they affirmed their gender were particular sources of internal stress.

Any time I'm in the presence of someone who knew me before—so, past family members, past friends, anybody like that—it's almost like every muscle in my body is in a heightened state of alert and my blood pressure, I can almost feel it pumping. Every word they say, every word they don't say, every flick of their eyes, everything they do is something that I'm focusing on and reading… So, constantly, I'm ready for them to say the wrong pronouns or use the wrong name, or speak to me in some negative way even if they haven't done it or aren't going to. Usually, by the time that situation is over, whether or not anything bad happened, I'll just burst into tears or just decompress emotionally after it's done because I'm so ready to be hurt, expecting to be hurt. (32, White, female/woman)

#### Could happen if or when I do not “pass” or “blend.”

Although representing a small proportion of the sample, several participants reported that they might expect rejection based on how well (or poorly) they “passed” or “blended” while in public. In presenting earlier in their gender affirmation process, or without the benefits of masculinizing or feminizing hormone therapy, participants perceived that others might react negatively more frequently.

When I first transitioned and didn't quite pass as well, I was worried about it [expecting rejection]. (25, Latino, MTF)

Anybody who's going through the transition, especially during the early phases, when you're getting “ma'am” half the time and “sir” half the time, you definitely notice stares… I don't deal with it so much anymore. I've been on hormones for the better part of a decade, so normally I don't have to worry about it too much. (31, White, MTF)

### Thoughts and feelings associated with expecting rejection

#### Fearful, worried about safety, and hypervigilant

Participants reported that the expectation of rejection often is associated with distinct feelings of fear and worry for their personal safety. Nearly universally, participants shared a common concern for the possibility of being a target for discrimination and victimization as a result of their gender identity.

I'm hypervigilant constantly. I think it's impossible really to be a trans female, and probably to be a trans male as well, and not be hypervigilant about what's going on around you—the people around you, and how they're acting and interacting with you and with each other, because violence creeps up on you really quickly and people are jerks. They're just f***ing a**holes and you never know when something is gonna happen, and so you always have to just be aware of what's going on. And it's a luxury that I think a lot of my cis friends don't realize they have, especially some of my White gay male friends. They don't get it that they can just walk around and not worry about being attacked or harassed. They always counter with, “Well, I got called a fag once.” And I'm like, “Okay, yeah, once. Try that every day.” (30, multiracial, MTF)

In addition to the fear of violence and discrimination, participants also reported that they often were on alert or on guard, and ready to react to threats from others.

To get to work, I have to go to a bus stop that's a mile away, so I have to walk a mile alongside the highway. I walk down that every day, and there's a stretch where these creepy guys hang out, and sometimes they'll yell things at me and I get catcalled a lot, and that kind of makes me on edge. I think it's pretty unlikely anything bad would happen but I think if, say, someone groped me and realized I was transgender, I think that could end pretty badly. So, I'm a little more hypervigilant in that case. …Usually, [when in this situation] I'm thinking about how fast I could run and I'll play out scenarios in my head. (25, Latino, MTF)

#### Anxious, stressed, and nervous

Every participant stated that they, to varying degrees, experienced anxiety and stress in association with the expectation of rejection. Participants described the internal stress as overwhelming, crushing, and awful.

So I recently went to a doctor in [home city] for the first time—nothing related to my transition at all, just a medical problem that I was having. Literally four or five hours before I went, I just felt really nervous or anxious about going there and just being like, “What if this happens? What if that happens?” Nothing happened. The doctor was really understanding, but it's just that preemptive stress, I guess. (25, multiracial, transsexual)

#### Physically and mentally exhausted by the end of the day

A marginal portion of participants shared that the process of managing the internal stress, over the course of the day and in different contexts, was physically and mentally taxing.

Well, there's definitely stress. Because you're constantly worried about everyone else and it's no wonder you have a hard time when you're constantly worried. So, yeah, there's worry, stress, and exhaustion. It's definitely tiring. (25, White, female/woman)

#### Depressed, self-loathing, and I am at fault

In addition to fear- and anxiety-based internal processes, participants also reported mood disturbances associated with the expectation of rejection. The primary experience centered on feelings of sadness and depression.

You feel like you're always on your guard and it's hard to get your hopes up about meeting someone new because, a lot of the time, they're going to act negatively without even getting to know you. So, it's depressing, in a way. And I think a lot trans people including myself, have dealt with depression, so it can be a little overwhelming. (31, multiracial, genderqueer)

In addition, participants detailed the ways in which they experienced feelings of shame and embarrassment, and negative thoughts about themselves.

It [expecting rejection] makes me sad. Like, I'm always going to be an alien—like an alien amongst friends. It's a lot of things like that. I tend to internalize a lot of stress and sadness, and wonder about myself rather than thinking so much about the people who stress me out. (32, White, female/woman)

#### Angry or frustrated with the situation and others

Although participants described the experience of expecting rejection generally as stressful and disheartening, they also voiced strong feelings of anger, frustration, and disappointment over the idea of expecting rejection from others.

There's a lot of anxiety and it's usually coupled with anger, which is maybe how I get through it. Like, if I have to use a public bathroom and I don't have another choice, I kind of get a little angry and it helps me to just do what I have to do. (26, Latino, masculine)

Notably, several participants expressed that expecting rejection, especially in retrospect, was sometimes a confusing process.

I honestly feel sorry for these people, that they can't just be open and accepting of everybody around them… I don't understand. I don't get why they feel a need to do this. (30, White, female/woman)

This was especially apparent when participants felt threatened or misunderstood by other minority populations (i.e., cisgender lesbian, gay, and bisexual) that, as the participants perceived, might experience similar minority stressors.

The gay community refuses to accept trans men and gender fluidity, and the lesbian community will sometimes become violent against trans women, and then they turn around and complain about the same things being done to them by the rest of society. It's like, “How can you cry about this when you're doing it to the rest of us?” (40, White, gender fluid)

#### Not supported, ignored, and rejected

Finally, participants additionally reported that, when experiencing the expectation of rejection, they sometimes believed that they were not supported by others and ignored.

I want to be treated like anybody else, so anticipating being an outsider isn't a great feeling. It's pretty upsetting, hoping that somebody would go up to bat for you, right? … It's like the one kid who gets punked on or bullied, and they kind of know that they're different, but hopes that other people will stand up for them, be nice to them, and fight for them, you know? (28, multiracial, male/man)

Some participants reported that they felt rejected by society.

I would get people that would look at me and talk to me like, “Is this freak really speaking to me?” instead of just talking to me like I'm a normal human being … And I even experience that while being in the presence of my wife. People would speak to her and think that it wasn't important to speak to me, although I'm standing there next to her. (34, Black, male/man)

### Coping strategies used to manage the expectation of rejection

#### Avoidance or escape

When expecting rejection and experiencing intense distress, participants stated that they frequently responded by engaging in avoidance strategies. Based on the participants' report, this occurred in two ways. First, participants reported that, if expecting rejection, they would avoid certain situations altogether.

Avoidance is a big thing that I do. I will avoid going to the doctor, even if I need to go to the doctor, or avoid going to the gym since I don't want to deal with locker room situations. (28, multiracial, FTM)

As a second method, participants noted that, if they did not have the immediate option to avoid the situation or were already in a potentially threatening setting, they used developed behavioral routines as a way to avoid becoming a target.

I usually try to hide in the [restroom] stall. I look around a lot when I'm inside and, if there's anyone in there, I try to hurry so they can't see me; or, I'll wait in there until there's no one else in the restroom and then rush out and wash my hands, and then escape. It's like, “I don't want to deal with this. I just want to go to the bathroom like a normal person and go on with my day,” you know? (31, multiracial, genderqueer)

When I go to the gym, I always keep earphones in the entire time because I just don't want to hear what's going on around me. I don't look up—my eyes are always three feet in front of my feet—I don't talk to anyone, and I don't want to hear the comments. (40, White, gender fluid)

#### Substance use

Participants reported that they would use substances in response to the stress associated with expecting rejection. Specifically, a third of the total sample reported alcohol use and smoking cigarettes as common coping strategies. Participants also mentioned marijuana, prescription drugs, and other unspecified substances used.

There are lots of things that I do that are probably not healthy for me to deal with the stress of having to navigate the world as a trans woman. I smoke cigarettes. That's one of my big coping mechanisms when I'm out in public. I don't really drink but I do smoke a lot of pot. That's honestly one of the main ways that I deal with stress that everyone and everyday life causes me—to escape some of the crap that's going on with being trans and escape some of, you know, the fears and issues that I'm gonna go through. (30, multiracial, MTF)

#### Cognitive or emotional coping strategies

A small proportion of participants detailed that they responded to the stress of expecting rejection by ruminating on what could occur, or what had already happened in the past.

I might think there's this tiny chance someone would react violently if they found out. So, I guess that's kind of in the back of my head sometimes… I guess the most unhealthy thing I did about that was ruminate on it. (25, Latino, MTF)

In addition to rumination, other participants noted that they might respond by becoming even more infuriated with the situation.

Whenever I get stressed out [about expecting rejection], I let go of my stress by being angry. I tend to lash out. …It always ends up being more harmful right? You always regret that. (28, multiracial, male/man)

### Intersections of race and ethnicity with expecting rejection

#### Being a person of color increases the expectation of rejection and helps you to prepare for the rejection

When asked to consider how the participant's race and ethnicity might impact their expectations of rejection, participants were clear in their general belief that being a person of color increases the risk for discrimination and violence. Others offered the notion that experiencing discrimination as a person of color helps prepare individuals to experience discrimination as a TGNC person.

There are definitely situations in which I might not even be thinking about my gender identity, but I'll expect to experience some form of discrimination or some sort of danger just based on my racial identity. (26, Latino, masculine)

I think the experiences that I had when I was young gave me a little bit of a thicker skin, and already taught me some things about diversity, and there's a wide array of responses to diversity. (34, multiracial, FTM)

Although participants of color readily detailed their own experiences of managing the internal stress associated with a dual minority status, White participants, given the frequency and detail of their responses, appeared to have little difficulty recognizing and acknowledging these same stressors.

I'm terrified of the threat of violence against me, knowing that if I'm ever raped that the chances of being beaten or murdered is way higher for me than others. But also knowing that it's not anywhere like it is for other girls who have darker skin than me. (32, White, female/woman)

#### Being White comes with privileges and decreases the expectation of rejection

Participants, quite commonly, discussed that being White comes with distinct social privileges and protections.

Well, I believe that I end up with White female privilege, which certainly has an advantage over, for example, being a woman of color. I know that I've had probably more opportunities than some women of color, especially some trans women of color that I know. (28, White, female/woman)

Notably, the notion of “passing” as White was salient to many participants, especially given that a large percentage of the sample was multiracial or multiethnic.

Every single trans person I know, who is a trans person of color, all of them have said that they're treated worse based on their race and being trans. So, I can only imagine that if I actually were perceived more regularly as a person of color, the discrimination that I would experience would be more so. (30, multiracial, MTF)

## Discussion

This study aimed to assess the saliency of expecting rejection by identifying how, and to what extent, expecting rejection manifests in TGNC individuals, and further clarify how they might respond to the expectation of rejection. Through in-depth interviews, study findings offer evidence that proximal stress is a salient experience for TGNC individuals. Despite the significant heterogeneity present in the sample, in terms of the unique experiences of individuals from different geographical settings, different racial and ethnic backgrounds, at different points in their gender affirmation process, and at different ages, study findings converge in a stable narrative. This convergence is quite meaningful and provides strong support that expecting rejection is a common experience for TGNC individuals, especially given the frequency with which it was reported. In addition to frequency, the severity of the experiences that participants reported was profound.

Qualitatively, participant narratives were punctuated with a sense of urgency, distress, and resignation. In other words, they shared stories about expecting rejection in ways that suggested that the experiences were intense and often life-threatening (e.g., experiencing fear, anger, and hypervigilance); upsetting and disparaging (e.g., experiencing frustration, sadness, and shame); and an expected part of their existence (e.g., thinking about the possibility of rejection in most contexts and with most people). Given these findings, it appears that the proximal stress experiences of TGNC individuals are consistent with the expecting rejection construct represented in Meyer's Minority Stress Model.^12,13^

Furthermore, these findings offer evidence regarding the largely theoretical association between expecting rejection (or felt stigma) and psychological distress. Consistent with the few published studies that have found expecting rejection to be positively associated with elevated levels of psychological distress,^11,21^ the current qualitative study provides context for why and how distress might develop. Generally, participants reported that their proximal stress experiences were cognitively and emotionally distressing. As the narratives detailed, TGNC participants specifically reported fear and anxiety, sadness and anger, and mental exhaustion, and perceived that they were ignored and alone in their experiences.

Although qualitative studies are not designed to assess for directionality among constructs (i.e., internal stigma leading to psychological distress), nearly universally, the participants in this study reported narratives that offer evidence that proximal stressors appear associated with health and well-being. Future research using a quantitative methodology should further explore the association of these constructs in a larger sample.

Beyond experiences of depression and anxiety already reported in the literature (and implicated in this study), TGNC participants indicated that experiences of fear, worry, and hypervigilance were highly prevalent. Given that these fear-based responses are often observed in individuals with a trauma history, it is important to further assess to what extent proximal stressors might match the level of severity and impact of distal stressors. Intuitively, and consistent with published literature, one might assume that distal stressors such as discrimination and victimization are the primary factors that promote traumatic stress in TGNC individuals. Yet, these findings suggest that proximal stressors might operate similarly. In addition, many participants reported that they heard stories of violence and discrimination from TGNC peers, which could be a form of vicarious trauma.^26^

From an intervention perspective, understanding the impact of internal stressors may inform clinical and public health approaches to trauma, which extends beyond what is happening to TGNC individuals that can be easily seen and measured (e.g., experiences of violence and discrimination), and focus on experiences that might be more covert and internalized (e.g., expecting and normalizing violence, or internalizing messages of shame and embarrassment).

Another consideration noted in the limited published literature is the use of avoidance strategies by TGNC individuals as a way to manage the expectation of rejection.^22,23^ Current findings are consistent with what has been previously reported. Generally, TGNC participants described situations that include gender markers or a clear gender binary system, that is, sex-segregated spaces, as being incredibly stressful, especially public restrooms. Although the fear of being outed was previously suggested as an indicator for why a TGNC individual might avoid situations,^22^ participants in this study detailed further concerns. Beyond simply being outed, participants expected confrontation (e.g., asked invasive questions or told that they were in the wrong restroom/locker room), harassment (e.g., catcalled or laughed at), discrimination (e.g., not welcomed at social events or differential treatment), and violence (e.g., physical and sexual assault). Therefore, avoidance strategies (i.e., situational avoidance) might actually represent an adaptive approach to mitigating proximal stress for TGNC individuals.

Although previous studies indicated the types of settings that TGNC individuals perceive as threatening,^22^ current study participants identified settings and also detailed ways in which they assessed for potential threats. Participants stated that they often thought about the experiences of other TGNC individuals who reported discrimination or violence specific to a particular context (e.g., public restrooms, work settings, or bars and clubs). Based on this information, participants reported that they would consider the possibility of a similar aversive experience, were they to be in the same context. Other participants stated that they often were alert and on guard in certain situations, paid attention to their surroundings, and actively looked around and assessed how others were responding. A few participants reported that they would consider how easily they could escape a given situation, were there to be a potential threat. Thus, participants not only assessed for perceived and real threats to their safety and dignity but also, additionally, some rehearsed how they might react. Participant narratives, therefore, support Meyer's model regarding the manifestation of hypervigilance when expecting rejection for TGNC individuals.^12,13^

Participants discussed strategies for managing the expectation of rejection when they could not avoid certain settings. Participants detailed routines that they developed to increase safety, including not making eye contact with others and remaining in restroom stalls for an extended period of time if someone was present and quickly exiting the space when no one was around. Other participants noted that they would enter certain settings with a friend to increase their sense of safety. In sum, the social interactions of participants appeared thoughtful, planned, deliberate, and skillful, reflecting the general stress associated with navigating social spaces as highly stigmatized and readily targeted individuals.

Notably, participants in this study discussed ways in which race and ethnicity intersected with the expectation of rejection. According to minority stress theory, it is possible for individuals to experience stressors specific to different minority identities.^12,13^ Therefore, individuals with multiple minority identities can, and likely, experience proximal stressors based on one of the minority identities or as a combination of several identities.

Accordingly, participants who were people of color stated that a dual minority status—specifically, being TGNC identified and a person of color—compounded the stress associated with expecting rejection. Participants of color readily discussed their experiences and perceived that expecting rejection (e.g., with the police, with employers, and when in unknown environments) was more frequent for them compared to White TGNC individuals. White participants noted similar discrepancies based on what they had seen and how their own treatment was differentiated. The reported stress of a dual minority status described by TGNC participants warrants further research and evaluation.

In addition, and perhaps as an indication of resilience (or as an unfortunate form of habituation to discrimination), participants of color frequently described that they were prepared for the possibility of gender-related rejection from others based on previous experiences of discrimination as a result of their race or ethnicity. Conversely, participants reported that being White or passing as White (as stated by several individuals of mixed race/ethnicity), brought many social privileges that reduced the expectation of rejection (e.g., being perceived as more valuable, more trustworthy, and not “standing out” when in public). Future research would benefit from exploring how a dual minority status might contribute to potential protective factors or enhance resilience when faced with stressful experiences. In addition, there is a need to understand how identity salience, as noted by Meyer,^12,13^ might impact the aforementioned. For example, research might investigate whether there are differences in the stress associated with racism depending on the salience of gender identity and/or stage of gender affirmation.

### Limitations

This study is not without limitations. The function of qualitative research is to more thoroughly understand unique phenomena, and develop theory from the perspectives and life experiences of the specific population. This is especially true for “hard-to-reach” populations, including TGNC individuals. However, given smaller sample sizes and methodological focus on reaching thematic saturation, qualitative research typically is not generalizable with any degree of certainty. In addition, participants' social desirability and recall bias likely impact the results—which is to be further considered in the context of any biases brought by investigators (e.g., the majority of the research team identifies as cisgender). Also, participants volunteered to be part of the research study, which likely contributed to selection bias (e.g., volunteerism). Similar to cross-sectional studies, and as previously noted, directionality cannot be determined from these data (e.g., experiencing the proximal stressor leads to specific thoughts and emotions, which then lead to specific coping strategies and subsequent mental health outcomes).

Recruiting participants through the Internet also presents challenges. Although previous research has outlined how to effectively recruit transgender participants and collect valid data through rigorous online methodological designs, Internet-based studies exclude participants who do not have access or the necessary resources to be online and likely represent a more narrowly focused group of participants,^27^ which could include an overrepresentation of White individuals. The benefits of using the Internet to recruit participants from geographically diverse locations are mitigated by the limitations of having a group of participants who might have very different and heterogenous life experiences.

Finally, this study only presents findings about the ways in which participants coped with expecting rejection, which were generally “negative.” Participants also noted many ways that they coped with general stress that demonstrated resilience in the face of this adversity, which was not discussed herein (an additional, forthcoming article will focus on general coping, strengths, and resilience in this sample—a separate aim of the parent study).

## Conclusion

This research represents one of the few studies designed to qualitatively investigate proximal stressors in TGNC individuals. These data demonstrate the extent to which expecting rejection might operate as a pervasive daily experience in the lives of TGNC individuals. Furthermore, this study provides evidence regarding the adverse impact of expecting rejection, as shown by the stressful cognitive and emotional responses reported by participants. Although emerging research has shown the deleterious impact of distal stressors (i.e., violence, discrimination, and stigma) on the health of TGNC individuals, the current finding demonstrate that proximal stressors likely have a similar devastating impact.

Given the frequency with which proximal stressors were reported by TGNC participants—and the reported experiences of fear, hypervigilance, sadness, and anger, in particular—there is a clear need and urgency to further evaluate the cumulative impact of the stress over time and identify future targets to intervene upon to mitigate potential harms. This is especially relevant given the current cultural climate in which TGNC individuals continue to remain targets for violence and discrimination—which has resulted in the murder of countless TGNC individuals worldwide, and the suicide of individuals who can no longer withstand the experience. Future research, including clinical intervention development and testing, should begin to prioritize the needs of this vulnerable population.

## Abbreviations Used

CQR: consensual qualitative research
FTM: female-to-male
MTF: male-to-female
TGNC: transgender and gender nonconforming

## Appendix: Interview Questions for Expectations of Rejection

### Expectations of Rejection

A lot of trans-identified folks mention that they have faced discrimination in their lives. Next, I'm going to ask you some questions about discrimination that you may have experienced by virtue of identifying as [GENDER IDENTITY LABEL].

1. First, I'm wondering: how you would define “discrimination?”

People can experience discrimination in many ways and in different contexts. One definition is that discrimination is ‘an event or process in which someone treats you differently—usually more negatively—because of the group, class, or category in which you belong.’

Whether or not transpeople have experienced discrimination themselves, many report that there are specific places—and interactions with specific types of people—where they feel certain they'll face discrimination.

1. Here I'm wondering: have you ever expected that you might experience discrimination because you identify as [GENDER IDENTITY LABEL]? For example, in preparing to go somewhere or to meet with someone, have you had the experience where you think, ahead of time, about the possibility of experiencing discrimination; or when you are out somewhere or with someone, you are thinking about the possibility of experiencing discrimination?

IF YES

• Tell me more about an experience that stands out to you the most when you expected to experience discrimination.
• A lot of people find the expectation of discrimination to be stressful or upsetting. If this is true for you, in what ways might that experience have been stressful or upsetting for you?
  ○ What were you thinking or feeling?
  ○ When people are stressed about something, they sometimes try to deal with it in different ways. We call this “coping.” If this is something you do when stressed about expecting discrimination, what are some ways that you coped with this or a related experience?

IF NO

• That's interesting. Most of the trans or gender nonconforming folks I've talked with have noted at least one time when they've expected to experience discrimination. Why do you think this might not be an experience that some trans or gender nonconforming folks face?

3. There's a term called “hypervigilance,” which means that we're on alert or on-guard when in certain situations—or all the time, even—but especially when we think that something bad could happen to us. Do you ever find yourself to be hypervigilant in certain situations, because you identify as [GENDER IDENTITY LABEL]?

IF YES

• Tell me more about that experience and what it was like for you.
• What thoughts or emotions did that bring up for you?
• We talked about coping earlier. What are some ways that you coped with this or a related experience?

4. Unfortunately, it is common for others—this can be friends, family, even people we don't know—to act or react negatively to people who are different from themselves in some way. What does it feel like to know and expect that other people whom you interact with might react negatively to you simply for being [GENDER IDENTITY LABEL]?

• What emotions come up for you as you think about the discrimination that you might face?
• What types of thoughts come up for you as you think about this expectation of discrimination?

5. What types of situations [PLACES, PEOPLE, ACTIVITIES] do you believe might lead transgender people to feel stress due to an expectation of discrimination?

• Why might that be?
• What about situations specific to you?
